# Challenges and opportunities on the utilisation of ionic liquid for biomass pretreatment and valorisation

**DOI:** 10.1038/s44296-024-00015-x

**Published:** 2024-05-15

**Authors:** Antonio Ovejero-Pérez, Pedro Y. S. Nakasu, Cynthia Hopson, Josiel Martins Costa, Jason P. Hallett

**Affiliations:** Department of Chemical Engineering, Imperial College London, South Kensington Campus, Exhibition Road, London, SW7 2AZ UK

**Keywords:** Crop waste, Chemical engineering

## Abstract

Biomass processing employing ionic liquids is already an established option at the laboratory scale. Ionic liquids can disrupt and deconstruct the lignocellulosic biomass network, giving rise to multiple options for valorisation. However, there is still much work remaining to accomplish the scale-up and commercialisation of ionic liquid-based biomass processing. Important issues such as ionic liquid cost and recyclability, among others, need to be carefully addressed. In addition, ionic liquids modify the structure and properties of the recovered materials, impacting potential applications. Due to the complex nature of ionic liquids, where multiple combinations of anions and cations are possible, these issues should be considered for each process and application, making it difficult to generalise for all cases. This perspective covers the main challenges and opportunities in the employment of ionic liquids for biomass processing, both in the biomass processing stage and in the valorisation of the recovered fractions. Among them, we discuss the importance of solvent recovery and costs as two critical issues to consider in biomass processing, as well as the major role lignin condensation plays in hindering ionoSolv lignin valorisation and different approaches to valorise the recovered cellulose.

## Introduction

Ionic liquids (ILs) are salts with melting points below 100 °C formed by the combination of different cations and anions. They exhibit excellent thermal stability, medium-high polarity, and negligible vapour pressure^1^. Their environmental friendliness makes them a suitable substitute for hazardous, unstable or flammable solvents in industrial applications. There are two main groups of ionic liquids: aprotic ionic liquids (AILs) and protic ionic liquids (PILs). The main difference between them is the presence of a free proton in the PIL structure^2^. This gives them a mild-to-strong acid character in contrast to AILs, acting like acid catalysts in the cleavage of lignin intramolecular and lignin-sugar bonds. PIL synthesis is simpler than the AILs synthesis, normally a neutralisation reaction between an acid and a Brønsted base, thus defining the acid-base ratio of the PIL, which is one of the main design parameters^3^.

Within the context of biomass processing, there are two main approaches. The first one aims to dissolve the whole lignocellulosic biomass structure to then recover the pretreated biomass, improving the subsequent yields by this solubilisation/precipitation process^4^. The second approach is to selectively dissolve lignin and hemicellulose, leaving the cellulose (practically) unaltered, which gives rise to the potential valorisation of the different biomass fractions separately. The latter is called the ionoSolv process^4^. The interplay between anions and cations holds significant importance in the effective solubilisation of lignin and cellulose^5^. For example, dialkylimidazolium-based ILs with strongly coordinating anions have shown notable dissolving capacity for lignocellulosic biomass and have displayed efficient pretreatment capabilities regarding delignification and fermentable sugar release^6^. However, the attention of researchers has recently focused on the toxicity, biodegradability, and biocompatibility characteristics of ILs. In this sense, biomass-derived components, such as amino acids, carbohydrates, and phenolic compounds, hold significant potential for sustainable delignification processes^7^. In this regard, choline-based ILs exhibited lower toxicity compared to dialkylimidazolium-based options, in addition to higher biodegradability in wastewater^8^. Cations based on phenolic compounds, such as vanillin and *p*-anisaldehyde, also allow the synthesis of ILs via reductive amination. In addition, ILs with carbohydrate-derived anions, such as 5-hydroxymethylfurfural or levulinic acid, have been used to pretreat biomass^9,10^. Although the efficiency of pretreating biomass with ILs is well known, few methods have been commercialised for ethanol production, in contrast with dilute acid and steam explosion. Thus, IL-based pretreatment technologies still face technoeconomic challenges that must be overcome before large-scale implementation^11^.

Lignocellulosic biomass is a plentiful and sustainable source of organic compounds that can be employed for bioenergy or biobased chemicals production. However, it is still underutilised, and its exploitation is mostly based on burning waste, often polluting the environment. Therefore, fractionative pretreatment is an attractive option to process lignocellulosic biomass and obtain biofuels and value-added products, usually by improving surface accessibility for enzymatic hydrolysis. Pretreatment also separates the cellulose and hemicelluloses from the lignin^12^, which can then be recovered and used, improving the economic feasibility of the process by lignin valorisation^13^. Furthermore, there is little to no production of inhibitors, acidic or alkaline substances (and what is produced is often extracted into the IL, providing a “free” detoxification). Some of the biofuels obtained from lignocellulosic sources are bioethanol, biomethanol, biobutanol, biodiesel, and biogas, among others. Biomass can also be utilised for obtaining value-added products, including organic acids, biochar, sugar, bio-oil, phenolic compounds, cellulose composites, hydrogels, and fragrances, among others^14–17^. This wide range of products makes biomass utilisation with ILs interesting to various sectors, such as the food, cosmetics, and pharmaceutical industries, showing a high potential for valuable by-products and promoting a circular economy. This perspective review covers what we think are the main challenges to overcome, both in terms of biomass processing and biomass fractions valorisation, in order to make biomass processing with ILs a reality at higher scales than laboratory scale, and thus providing a new vision and approach when thinking about treating biomass with ILs.

## Challenges of ionic liquid pretreatment

### Biocomponents and biomass solubility

Pretreatment or fractionation is a crucial stage in converting lignocellulose into valuable products. ILs are involved in both the pretreatment of biomass and its transformation into valuable products, with the dissolution of the biomass structure (part or whole) playing a crucial role in both cases. Softwood biomass presents a higher lignin content compared to hardwoods and grasses, and this lignin is mainly formed by guaiacyl units. On the other hand, lignin from hardwoods and grasses is formed by syringyl and guaiacyl units (and *p*-hydroxyphenyl units to some extent)^4^. This complex structure and varied composition of biomass makes dissolution challenging^4^. Biomass dissolution relies on disrupting hydrogen bonds among lignocellulosic components, with the anion forming new hydrogen bonds, particularly with the cellulose hydroxyl groups. The cation’s potential role in polymer dissolution is linked to its size and hydrophobic properties^18^. The extent of cellulose dissolution directly impacts the effectiveness of the overall process, providing an appropriate reaction medium for cellulose derivatization and directly correlating with the yield of end products. Incomplete solubility not only affects yield but also complicates product extraction. An ideal solvent would facilitate maximum reactant solubility, support the desired chemical transformation, and aid in the easy recovery of the final product. Consequently, selecting the optimal IL system tailored to both the feedstock and intended transformation remains a crucial focus for future research and advancements^11,15,19^.

### Ionic liquid recovery and recycle within a biorefinery context

IL recovery and reuse is paramount in IL processing of lignocellulosic biomass since ILs are more expensive compared to traditional solvents such as water or ethanol, which limits their industrial application^20^. IL pretreatment performance can be compromised by the presence of impurities, including soluble lignin particles and/or carbohydrate degradation products such as furfural or HMF. Additionally, IL thermal stability can also be problematic, making their reuse a significant challenge^21^. In theory, successful IL recycling requires complete product recovery, impurity elimination, and long-term IL stability. However, this does not occur when lignocellulosic biomass is solubilised, especially regarding the presence of more pH-sensitive biopolymers such as proteins and hemicelluloses. One strategy to overcome this is to perform a pre-extraction of the proteins and/or hemicelluloses to ensure this fraction will be properly valorised. An additional step also means extra unit operations will be considered in the process, which can increase the process CAPEX.

The water usage for the washing step after pretreatment demands high volumes of water. Nakasu et al.^22^ showed that a balance must be reached on the minimum amount of water that can be used to achieve high yields in the enzymatic saccharification while also recovering the majority of the protic IL monoethanolammonium acetate, [MEA][OAc]^22^. High volumes of washing water also result in high energetic requirements for water evaporation. Silva et al.^23^ showed that despite high energy intensity, the recovery of technical lignin increases the yield of products and the net revenue of the biorefinery^23^.

**Fig. 1:**
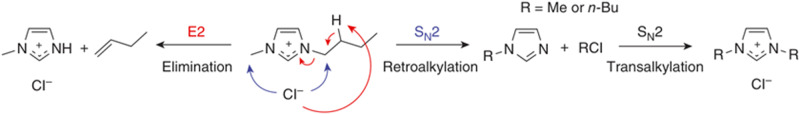
Decomposition pathways of [Bmim]Cl (adapted from ref. 25).

It is also difficult to generalise the potential of IL recovery and reuse because of the multitude of possible cation and anion combinations. Recovery potential also varies with IL type. For instance, two aprotic ILs, 1-allyl-3-methylimidazolium chloride ([Amim][Cl]) and 1-butyl-3-methylimidazolium acetate ([Bmim][OAc]) were tested as pretreatment agents of eucalyptus^21^. The first IL, [Amim][Cl], afforded lower saccharification yields, but it showed higher recovery rates (>90% compared to <80%) compared to ([Bmim][OAc]). On the other hand, Brandt-Talbot et al. (2013) reported IL recoveries of 99% with a protic IL, such as [TEA][HSO_4_]^24^. Sklavounos et al.^25^ mentioned that a general trend regarding IL thermal stability is correlated with the basicity of the anion, with more basic anions lowering the IL thermal stability. IL decomposition pathways may occur through E2 elimination of the alkyl group or S_N_2 attack at imidazolium alkyl positions (Fig. 1). Other decomposition pathways can also take place, including anion dissociation^25^.

There are several methods to recover ILs, including distillation, liquid-liquid extraction and the use of kosmotropic salts (to induce the formation of aqueous biphasic systems)^25^. Distillation is a common method for IL recovery due to its high thermal stability, but it can be energy-intensive and depending on the ionicity of the IL (i.e., more molecular than ionic), the acid-base ratio may be altered. Liquid-liquid extraction and membrane separation are eco-friendly alternatives for IL recovery, achieving high efficiency, but they can be problematic regarding lignin adsorption. Kosmotropic salts such as K_3_PO_4_, K_2_CO_3_, and Na_2_HPO_4_ may be used to recover ILs such as [Emim][OAc] in a strategy similar to that of Shi et al.^26^. Economic viability and environmental impact are crucial considerations when choosing a recycling method, with a 97% or higher recovery yield being economically favourable at an IL price of 2.5 $/kg^27^.

### Metal corrosion due to ILs

Choosing the materials of construction for a chemical process is critical for safety and economic reasons, especially with ionic liquids, as they are salts with, often, acidic pH in an aqueous solution. However, very little is still known about IL corrosivity and how it occurs, as the majority of the studies regarding IL employment in biomass processing are focused on laboratory scales rather than industrial applications. However, corrosion can lead to structural and equipment failure and, therefore, to catastrophic consequences^28^. For example, in the year 2020, it was estimated that corrosion costs worldwide approximately 2.5 trillion $US per year^29^.

There are different methods to estimate the corrosion rates (CR), with gravimetric methods being the most employed ones, where CR is estimated from the weight loss of a metal immersed in an IL for a certain period of time^30^. However, this presents some limitations, such as not providing information about the corrosion mechanisms. It also assumes that the surface area remains constant with corrosion, which is not always the case, leading to inaccurate CR estimation. Recent studies have focused on discussing CR as a 3D phenomenon, taking into account the surface area changes^31^.

It is expected that material corrosion will be a critical challenge for the industrial application of ILs, as they are salts with medium to high conductivity, and that are normally employed at high temperatures, which could potentially increase the CR. For example, even with non-acidic or mildly acidic anions such as [Tf_2_N], [PF_6_] or [Cl], CR was between 5.6–13 µm/year^32^. Increased temperatures cause an increment in CR, i.e. CR increased sixfold from 20 to 70 °C with [Tf_2_N]-based IL^33^. However, the information about metal corrosion in ILs is limited despite the number of publications regarding IL applications in biomass processing and needs to be considered since it is very often forgotten at a laboratory scale (especially when glassware is used). In addition, their chemical heterogeneity, with a great number of anions and cations combination, makes it difficult to understand corrosivity, and each IL needs to be tested in this regard.

### IL cost

Many of the ILs that are employed in biomass pretreatment have high synthesis costs, which is considered one of the major drawbacks of their large-scale use. Most ILs require a multitude of synthesis and purification steps, which would make them prohibitively expensive at an industrial scale. The price of aprotic ILs, those commonly used in biomass processing, ranges from 2.50 to 50 $/kg, which is much more expensive than any commercial organic solvent^27^. These prices are directly related to the cost of the ionic liquid, as well as with their recovery and reuse and the development of technologies that enable their effective use^27^. Protic ILs, present large-scale production costs around 1–1.5 $/kg^34^ which is similar to bulk organic solvents such as acetone and toluene. The ease of synthesis also minimises the production steps involved, which reduces their environmental impact^35^. Thus, the effective application of protic ILs has been a major driver for the boost to the economic viability of biomass processing with ILs^36,37^. However, these prices are still higher than the prices of conventional solvents such as ethanol (0.49 $/kg)^34^. And, while it is true that other factors such as non-volatility or lower corrosion of materials (when compared to acid treatments) need to be considered^34^, it seems necessary to use low-cost and reusable protic ILs that can be easily recovered in order to implement biomass processing with ILs on a larger scale. An example of this is the company Lixea through their Dendronic process, successfully employing low-cost ILs to valorise biomass at a pilot plant scale^38^.

However, there are still few studies that address the economic feasibility of IL pretreatment, as the majority of ILs are currently synthesised at a laboratory scale, with prices much higher than the prices they would have on an industrial scale. Chen et al.^39^ proposed an equation for protic ILs cost estimation from the prices of the acid and the base^39^; however, it doesn’t take into account further purification steps. In addition, some of the acids and amines employed in protic IL synthesis (including alkylimidazoles) are not produced at a large scale, so their prices are not consistent or mature. This could also create supply chain issues, especially when considering the production of imidazolium-based amines (which are specialty chemicals produced at a scale of a few tonnes per annum) vs alkylamines, some of which are produced at a commodity scale (hundreds of thousands of tonnes per annum). Employing novel or specialty amines (such as alkylimidazoles) would therefore require a scale-up of the amine (cation) synthesis even before the IL. All of this makes IL cost estimation an important, and very often difficult, step on the scale-up and design of an IL-based biomass pretreatment process; as with corrosion, it is often not considered at the laboratory scale.

## Challenges in the valorisation of biomass fractions from IL treatment

### The sugar platform under the IL biorefinery perspective

The impact of IL pretreatment on the fractionation of lignocellulosic biomass can be roughly divided by the nature of the anion; they can be neutral but are more often either alkaline or acidic^4^. Alkaline ILs derive from weak Bronsted acids such as acetic or formic acid, and they tend to solubilise most of the lignin and partly solubilise the hemicellulosic fraction, leaving a pulp rich in cellulose and hemicellulose. Upon enzymatic hydrolysis of the pulp, a syrup rich in C5 and C6 sugars is produced that can be metabolised into bioethanol by either engineered *Saccharomyces cerevisiae* or wild-type yeasts such as *Scheffersomyces stipitis, Spathaspora passalidarum and Kluyveromyces marxianus*^40^. Other fermentation bioproducts can be produced from this mixed syrup, such as succinic acid and butanol, by other C5/C6 metabolising bacteria, such as from the genus *Actinobacillus* and *Clostridium*^41–43^.

Acidic ILs present extra dissociable protons, such as the case of triethylammonium hydrogensulfate, [TEA][HSO_4_], or 1-ethyl-3-methylimidazolium chloride with excess hydrochloric acid, [Emim][Cl] + HCl. These ILs behave similarly to a dilute acid pretreatment by solubilising both lignin and hemicellulose fractions, leaving a cellulose-rich pulp that can be then hydrolysed by enzymes into a glucose syrup. The C6-rich hydrolysate can then be fermented by *Saccharomyces cerevisiae* into bioethanol or other glucose-metabolising microorganisms^40^. The main caveat of utilising acidic ILs is that it is difficult to recover solubilised hemicellulose sugars from the IL, especially because they tend to dehydrate into furfural, which is quite reactive and volatile. There are two strategies that can be used to overcome that: (1) a hemicellulose pre-extraction step via a milder pretreatment such as hydrothermal or liquid-hot water, or (2) direct furfural production during pretreatment in a similar way of conventional furfural production from grassy biomass such as corncobs or sugarcane bagasse. The former strategy has been explored by Ovejero-Pérez et al.^44^ and Rigual et al.^45^ on the pretreatment of eucalyptus and pinus, respectively, with hemicelluloses recovery up to 87%^44,45^. The second strategy has not been employed yet; instead, several studies based on biphasic (organic and aqueous) systems have been explored; however, they present a low prospect of industrial application due to the use of volatile organic solvents^46^.

Another interesting approach is to perform a one-pot IL pretreatment-enzymatic saccharification step or one-pot with combined IL pretreatment, enzymatic hydrolysis and yeast fermentation steps with biocompatible ILs^26,47^. The main advantage of one-pot processes is the reduction in unit operations between the steps (pretreatment, enzymatic saccharification and fermentation) which potentially decreases the CAPEX (capital expenditure related to acquiring, upgrading and maintaining equipment) of the process. Water usage is also lower, according to Shi et al.^26^. However, there are several issues that may arise from the one-pot approach: (1) enzymes and microorganisms need to be compatible with the IL both in terms of toxicity and osmoregulation, (2) IL biocompatibility may also mean the IL can be assimilated and metabolised by the microorganism which will consume it and therefore they cannot be fully recycled, and (3) pH adjustment for the enzymatic saccharification and fermentation is required and consumes strong acids such as sulfuric and hydrochloric acids. Therefore, the one-pot strategy, though extremely promising, still requires much development.

### Importance of lignin valorisation

Lignin has traditionally been used to obtain energy, mainly due to its heterogeneity and its difficult isolation without making it more recalcitrant, leading to poor lignin valorisation^48^. However, lignin is the major sustainable source of aromatic compounds, and it’s been proven that lignin valorisation can improve the economic and environmental competitiveness of biorefineries, leading to growing interest in increased utilisation^49,50^. For example, the valorisation of lignin extracted with ILs pretreatment could compensate for the cost associated with the introduction of this new technology. According to a simulation by Klein-Marcuschamer et al.^27^, the minimum ethanol selling price in an IL-based biorefinery could be lowered by 1.5 $/gal for every extra 1 $/kg that is added to the lignin selling price, so high-value added lignin applications should be considered^27^. It should be noted, however, that kraft lignin selling prices have never exceeded even $1/kg.

However, lignin from IL pretreatment usually presents difficulties for utilisation. It is normally highly condensed, with high molecular weights and heterogeneity, reaching polydispersity values between 10 and 70, especially high when the most acidic ILs are used^44,51,52^. This high heterogeneity could hinder lignin valorisation. One strategy to overcome lignin heterogeneity is lignin fractionation, making it possible to valorise lignin fragments of different molecular weights separately^53^. Another problem with lignin condensation due to the more acidic conditions of IL-based pretreatment is a high C-C linkage content. These linkages are more stable than C-O^54^, leading to difficulties in lignin utilisation as a source of low molecular weight aromatic compounds. However, the high C-C content and high molecular weights of these lignins could potentially lead to its application as a material reinforcement due to a higher thermal stability^55,56^. Unfortunately, these applications are not always of high value.

Although there are a lot of possible lignin applications, there has been little focus on the valorisation of lignin from IL-based biomass pretreatment processes. This knowledge gap is an interesting opportunity since lignin is considered one of the keys to an economically competitive biorefinery, especially if unconventional solvents, such as ILs, are involved^49,50^.

### Cellulosic materials

Cellulose pulps or cellulose-rich materials (CRM) from lignocellulosic biorefineries have grown in popularity in materials science due to high strength, environmental benefits and biodegradability. While cellulose presents a sustainable alternative to synthetic polymers, its complex crystalline structure and robust hydrogen bond network pose processing challenges. This resistance to traditional solvents hinders its modification and conversion. However, the scientific community sees potential in using ILs as a solution to these challenges, paving the way for advanced cellulose-based material production. Using ILs as solvents can yield a diverse range of cellulosic materials, including cellulose fibres, nanocellulose, ionogels and hydrogels, to name a few, and also the possibility to obtain cellulose derivatives such as phosphate or acetate cellulose, among others^57–62^. Many of them could be produced using cellulose-rich pulps with different amounts of lignin and hemicellulose, and not just highly pure cellulose pulp^63–66^. Each of these materials possesses distinct characteristics and is produced through specific methods, underscoring the adaptability of ILs in cellulose processing. The breadth of materials attainable using ILs showcases their potential and the versatility they bring to the table in the field of cellulose-based material formulation.

One of the main challenges with cellulosic materials and their future production at large scale is the inconsistent cellulose pulp solubility and the lack of information about the scalability of the overall process. The dissolution of cellulose pulp, which may contain varying levels of lignin and hemicellulose, is a pivotal step in the formation of cellulosic materials using ILs^57^. Extensive research is essential to either circumvent the use of bleaching processes—which are traditionally employed to eliminate lignin—the removal of the hemicellulose to improve the cellulose functionalization, or to develop novel ILs capable of simultaneously dissolving lignin and cellulose present in the pulp to obtain materials with both components. By doing so, a more environmentally friendly and efficient approach to processing cellulose pulp could be established, reducing the need for separate purification steps, and expanding the range of viable raw materials.

The production of cellulose fibres is the most technically advanced cellulosic materials process using ILs, via the Ioncell-F process^67^. It has some advantages against the traditional viscose (CS_2_) and Lyocell (NMMO) processes, making it very promising for an industrial application. The traditional viscose method, which employs hazardous substances such as CS_2_, poses a significant environmental concern due to the emission of pollutants including H_2_S, SO_2_, strong bases, and sulfuric acid^68^. Lyocell faces challenges arising from secondary oxidative reactions, thermal instability, elevated temperatures required for the dissolution process (~120 °C), and uncontrolled fibrillation when using the NMMO solvent, which makes the process more difficult to control at large scale^69^. The advantages of Ioncell-F process, such as a lower operating temperature (75 °C), higher tenacities in fibres, and homogeneous and dense fibrillar structure in the product^70,71^, are important enough to make the IL process viable.

The transition of cellulose-derived materials from laboratory research to industrial application is more complex than other processes. Regarding IL-based cellulosic materials, there’s a gap in research when it comes to upscaling production. Most studies focus on small quantities, offering limited insight into potential challenges, solutions, or cost structures at an industrial level (or even how materials properties will respond to a commercial fibre line). This lack of information can deter potential investors or industries from adopting the technology, fearing unforeseen challenges and expenses. To truly harness the potential of cellulose-based materials on an industrial scale, a comprehensive approach is vital. This involves not just optimising the processing techniques but also expanding research to address upscaling challenges, understanding the broader economic implications, and fostering collaborations between academia and industry to devise sustainable and cost-effective strategies.

In summary, although the path to incorporating ILs in cellulose material sourcing has challenges, the prospects are promising. Through persistent research and advancements, these obstacles can be surmounted, paving the way for sustainable, versatile, and efficient cellulose-derived materials across different sectors.

## Final remarks

Ionic liquids are promising solvents in the valorisation of biomass since they can dissolve and deconstruct the different fractions that form it, leading to a wide range of possibilities towards different applications. However, there’s still a lot to be done, especially when considering a scale-up of the biomass deconstruction process, where IL reutilisation, cost and corrosivity, for example, play a crucial role in the feasibility of the process. These things tend to be forgotten at a laboratory scale. Regarding the valorisation of the obtained fractions, the possibilities and challenges are even larger. Due to the IL heterogeneity, lots of different applications can be proposed, employing ILs in the biomass fractionation and even in the production step, as in the cellulosic materials formulation. It is important to consider high value-added applications for the isolated fractions due to the complexity of the IL and biomass systems; and consider the chosen application when studying the IL pretreatment process to tune the conditions if necessary to obtain a fraction with the desired properties to be employed. This is often forgotten when optimising processes at the laboratory scale. Now that the IL pretreatment process is well-known, it is expected that research will focus on exploring ways for valorising the recovered biomass fractions into high value-added applications, as well as on studying paths for process intensification to make biomass processing with ILs feasible.

## Data Availability

No datasets were generated or analysed during the current study.
